# Urinary cadmium concentration is associated with the severity and clinical outcomes of COVID-19: a bicenter observational cohort study

**DOI:** 10.1186/s12940-024-01070-6

**Published:** 2024-03-19

**Authors:** Li-Chung Chiu, Chung-Shu Lee, Ping-Chih Hsu, Hsin-Hsien Li, Tien-Ming Chan, Ching-Chung Hsiao, Scott Chih-Hsi Kuo, How-Wen Ko, Shu-Min Lin, Chun-Hua Wang, Horng-Chyuan Lin, Pao-Hsien Chu, Tzung-Hai Yen

**Affiliations:** 1 Department of Thoracic Medicine, Linkou Branch, Chang Gung Memorial Hospital, Taoyuan, Taiwan; 2 College of Medicine, Chang Gung University, Taoyuan, Taiwan; 3 Department of Thoracic Medicine, New Taipei Municipal TuCheng Hospital and Chang Gung University, Taoyuan, Taiwan; 4 Department of Respiratory Therapy, Chang Gung University College of Medicine, Taoyuan, Taiwan; 5 Division of Rheumatology, Allergy, and Immunology, Linkou Branch, Chang Gung Memorial Hospital, Taoyuan, Taiwan; 6 Department of Nephrology, New Taipei Municipal TuCheng Hospital and Chang Gung University, Taoyuan, Taiwan; 7 Department of Cardiology, Linkou Branch, Chang Gung Memorial Hospital, Taoyuan, Taiwan; 8 Department of Nephrology, Chang Gung Memorial Hospital, Chang Gung University College of Medicine, Linkou, No. 5, Fu-Shing St., GuiShan, Taoyuan 33305 Taiwan; 9 Clinical Poison Center, Center for Tissue Engineering, Kidney Research Center, Chang Gung Memorial Hospital, Taoyuan, 33305 Taiwan

**Keywords:** Cadmium, Nickel, SARS-CoV-2, COVID-19, Severity, Outcomes, Mortality

## Abstract

**Background:**

Cadmium and nickel exposure can cause oxidative stress, induce inflammation, inhibit immune function, and therefore has significant impacts on the pathogenesis and severity of many diseases. Severe acute respiratory syndrome coronavirus 2 (SARS-CoV-2) infection can also provoke oxidative stress and the dysregulation of inflammatory and immune responses. This study aimed to assess the potential associations of cadmium and nickel exposure with the severity and clinical outcomes of patients with coronavirus disease 2019 (COVID-19).

**Methods:**

We performed a retrospective, observational, bicenter cohort analysis of patients with SARS-CoV-2 infection in Taiwan between June 2022 and July 2023. Cadmium and nickel concentrations in blood and urine were measured within 3 days of the diagnosis of acute SARS-CoV-2 infection, and the severity and clinical outcomes of patients with COVID-19 were analyzed.

**Results:**

A total of 574 patients were analyzed and divided into a severe COVID-19 group (hospitalized patients) (*n* = 252; 43.9%), and non-severe COVID-19 group (*n* = 322; 56.1%). The overall in-hospital mortality rate was 11.8% (*n* = 68). The severe COVID-19 patients were older, had significantly more comorbidities, and significantly higher neutrophil/lymphocyte ratio, C-reactive protein, and interleukin-6 than the non-severe COVID-19 patients (all* p* < 0.05). Blood and urine cadmium and urine nickel concentrations were significantly higher in the severe COVID-19 patients than in the non-severe COVID-19 patients. Among the severe COVID-19 patients, those in higher urine cadmium/creatinine quartiles had a significantly higher risk of organ failure (i.e., higher APACHE II and SOFA scores), higher neutrophil/lymphocyte ratio, lower PaO_2_/FiO_2_ requiring higher invasive mechanical ventilation support, higher risk of acute respiratory distress syndrome, and higher 60-, 90-day, and all-cause hospital mortality (all* p* < 0.05). Multivariable logistic regression models revealed that urine cadmium/creatinine was independently associated with severe COVID-19 (adjusted OR 1.643 [95% CI 1.060–2.547], *p* = 0.026), and that a urine cadmium/creatinine value > 2.05 μg/g had the highest predictive value (adjusted OR 5.349, [95% CI 1.118–25.580], *p* = 0.036).

**Conclusions:**

Urine cadmium concentration in the early course of COVID-19 could predict the severity and clinical outcomes of patients and was independently associated with the risk of severe COVID-19.

**Supplementary Information:**

The online version contains supplementary material available at 10.1186/s12940-024-01070-6.

## Background

Coronavirus disease 2019 (COVID-19) is caused by severe acute respiratory syndrome coronavirus 2 (SARS-CoV-2) infection. The COVID-19 pandemic caused a devastating public health crisis, resulting in a substantial socioeconomic burden and significant morbidity and mortality worldwide, contributing to an estimated 7 million deaths as of October 2023 [1]. Individuals with predisposing risk factors may be prone to severe COVID-19, and about one-third of hospitalized patients with COVID-19 may develop severe hypoxemia complicated with acute respiratory distress syndrome (ARDS). ARDS can subsequently lead to multiple organ failure and death, and the mortality rate of COVID-19-induced ARDS has been reported to be up to 50% [2–4]. Therefore, identifying the risk factors and prognostic determinants for serious complications of COVID-19 is crucial to predict the severity of disease at an early stage, prevent deterioration, and decrease morbidity and mortality.

Essential and non-essential metals can exert nutritional or toxic effects and may contribute to excessive inflammation and impaired immune response in human body. A recent systematic review found that certain metals have either beneficial or harmful effects on COVID-19 outcomes. Zinc, selenium, and iron were shown to bolster the immune response and elevated levels were associated with reduced severity of COVID-19. Conversely, higher serum and urinary copper, as well as higher serum magnesium, were associated with increased severity and mortality in COVID-19 patients [5]. However, this literature review provided limited evidence regarding the positive or negative effects of non-essential metals, including cadmium and nickel, on COVID-19 outcomes due to the scarcity of studies.

Cadmium, a heavy metal toxicant, environmental pollutant and carcinogen, is harmful to immune function and multiple organ systems including cardiovascular, pulmonary, hepatic, renal, skeletal, and reproductive systems [6]. Cumulative cadmium exposure is a risk factor for the severity of infectious disease including respiratory viral infectious diseases (influenza virus and respiratory syncytial virus), non-airborne viral diseases, bacterial pneumonia, Helicobacter pylori, Toxoplasma gondii, and hepatitis B virus infections [7–10]. The primary source of cadmium exposure in the general population is through diet and cigarette smoking, with other routes including airborne pollution and occupational exposure [11]. The biological half-life of cadmium is extremely long in the human body, ranging from around 6 to 38 years. Cadmium is excreted mostly in urine, and urinary cadmium concentration can be considered an indicator of chronic exposure and total body burden of cadmium, whereas blood cadmium indicates recent acute exposure [12–17].

Cadmium exposure inhibits innate and adaptive immunity by increasing inflammatory cytokines and chemokines, dysregulating gene expression, and increasing susceptibility to microbial invasion, including viral infection. Cadmium can upregulate the generation of reactive oxygen species (ROS) and activate oxidative stress leading to cell apoptosis, tissue damage and eventually organ dysfunction [6, 16, 18–20].

Nickel, another environmental pollutant and carcinogen, can exhibit a range of toxic effects on human health including developmental toxicity, hematotoxicity, reproductive toxicity, neurotoxicity, genotoxicity, carcinogenicity, and immunotoxicity. Exposure to nickel primarily occurs through inhalation, ingestion of contaminated food, and dermal absorption from environmental or occupational sources. The half-life of blood nickel is approximately between 20 and 34 h. Nickel is primarily excreted through urine, exhibiting a half-life of urine nickel ranging from 17 to 53 h. Nickel is not a cumulative toxin, and urine nickel concentration only reflects recent nickel exposure [21, 22]. As an immunotoxic contaminant, nickel can promote ROS accumulation and suppress the antioxidant system, which leads to oxidative stress, mitochondrial dysfunction, DNA damage, and apoptosis [23–25].

SARS-CoV-2 infection can also lead to the excessive production of ROS, thereby inducing oxidative stress, endothelial dysfunction, persistent inflammation, and dysfunction of antioxidant defense mechanisms [26–28]. Therefore, cadmium and nickel exposure and SARS-CoV-2 infection can provoke oxidative stress and lead to dysregulated inflammatory and immune responses, which may then exacerbate the severity of clinical diseases with subsequent multi-organ injury and even death. The respiratory system is an important target for SARS-CoV-2 infection and cadmium or nickel exposure. However, the relationship between cadmium and nickel exposure and SARS-CoV-2 infection and their effects on the severity of COVID-19 have yet to be elucidated.

Therefore, the objective of this study was to assess the potential impact of cadmium and nickel exposure as assessed using blood and urine concentrations on the clinical presentations of patients with COVID-19.

## Methods

### Study design and patients

This study was based on retrospective analysis of COVID-19 patients who were hospitalized or outpatients with confirmed acute SARS-CoV-2 infection by positive real-time reverse transcriptase-polymerase chain reaction or antigen test results including home-based testing between June 2022 and July 2023 at Chang Gung Memorial Hospital (CGMH) Linkou branch and New Taipei Municipal TuCheng Hospital in Taiwan. Blood and urine samples were collected to measure the concentrations of cadmium and nickel within 3 days of the diagnosis of acute SARS-CoV-2 infection. The exclusion criteria were: (1) age < 20 years, (2) mortality within 3 days after hospitalization, (3) acute kidney injury with oliguria, (4) end-stage renal disease requiring maintenance dialysis, (5) reinfection with SARS-CoV-2, and (6) history of acute or chronic residential, occupational, or other exposure to cadmium and nickel when residing in a cadmium or nickel contaminated area or working in a cadmium or nickel emitting industry through electronic medical record review. The local Institutional Review Board for Human Research approved this study (CGMH IRB No. 202301066B0) and waived the need for informed consent due to the retrospective nature of the study.

### Definitions

The clinical spectrum of SARS-CoV-2 infection was categorized according to the severity of illness following the National Institutes of Health's Coronavirus Disease (COVID-19) Treatment Guidelines [29]. Severe COVID-19 was defined as shortness of breath, respiratory rate > 30 breaths per minute, blood oxygen saturation < 94% on room air at sea level, or a ratio of partial pressure of oxygen in arterial blood (PaO_2_) to fraction of inspired oxygen (FiO_2_) (PaO_2_/FiO_2_) < 300 mmHg [29, 30]. The enrolled patients were then stratified into severe and non-severe COVID-19 groups accordingly. The Youden index was used to determine cutoff values to categorize the patients into high or low blood and urine cadmium level groups. Patients in the severe group were classified by quartiles of blood and urine cadmium values at admission with the aim of exploring potential associations with clinical outcomes of COVID-19. ARDS was defined in accordance with the Berlin criteria [31]. Hospital mortality was defined as death from any cause during the hospital stay. Patients who remained alive for 90 days after discharge from the hospital were defined as survivors.

### Data collection

Demographic data, smoking status, underlying comorbidities, and laboratory data including interleukin-6 (IL-6), and cadmium and nickel concentrations in blood and urine samples were recorded for all participants. In addition, Acute Physiology and Chronic Health Evaluation II (APACHE II) score, Sequential Organ Failure Assessment (SOFA) score, the use of antiviral agents or other medications at the time of acute SARS-CoV-2 diagnosis, and type of respiratory support were also recorded. The dates of confirmed acute SARS-CoV-2 infection, hospital and intensive care unit (ICU) admission, shock status, use of inotropic agents, renal replacement therapy during the course of acute SARS-CoV-2 infection, the onset of ARDS, mechanical ventilator initiation and liberation, ICU and hospital discharge, and time of death were also recorded.

### Outcome measurements

The 28-day, 60-day, 90-day, and all-cause hospital mortality were evaluated. The incidence of shock status, use of inotropic agents, acute kidney injury, renal replacement therapy, ARDS occurrence, ICU admission rate, duration of mechanical ventilation, length of ICU stay, and length of hospital stay were recorded.

### Measurements of blood and urine cadmium and nickel concentrations

Blood specimens were collected in 6 mL plastic blood collection tubes containing K2EDTA as an anticoagulant (BD, Franklin Lakes, NJ, USA). Urine specimens were collected in 10 mL metal-free plastic collection tubes. Both blood and urine specimens were stored at 4 °C, and cadmium and nickel measurements were conducted using inductively coupled plasma mass spectrometry. The relevant details pertaining to the methods used for the cadmium and nickel measurements are supplied in Additional file 1.

### Statistical analysis

Continuous variables were presented as mean and standard deviation for normally distributed variables or median and interquartile range for non-normally distributed variables. Analysis of variance, the Kruskal–Wallis test, Student’s *t* test, or Mann–Whitney U test was used to compare continuous variables among groups. Categorical variables were reported as numbers and percentages and were compared using the chi-square test for equal proportions or Fisher’s exact test, and if the overall *p* value was statistically significant, the post hoc test was conducted in the next step. Receiver operating characteristic (ROC) curves and the Youden index were used to determine cutoff values to dichotomize continuous variables. Univariable analysis was used to identify the risk factors associated with severe COVID-19 in the first step, followed by the construction of multivariable logistic regression models with stepwise selection. The results were presented using odds ratio (OR) and 95% confidence interval (CI). All statistical analyses were performed using SPSS version 26.0 (IBM Inc., Armonk, NY), and a two-sided *p* value < 0.05 was considered statistically significant.

## Results

**Fig. 1:**
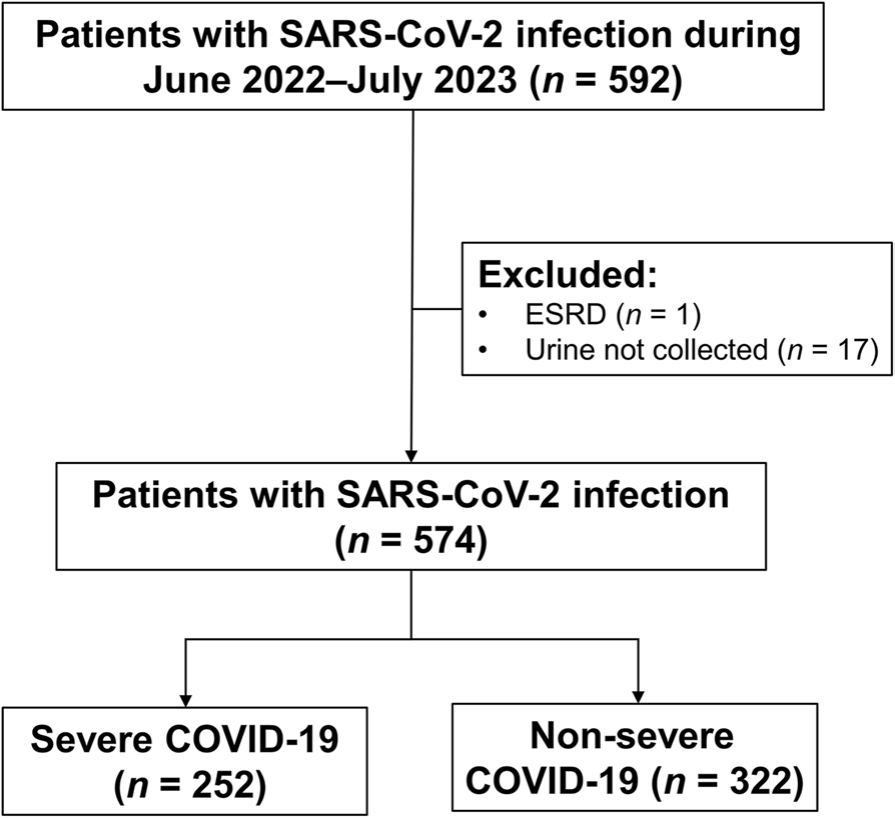
Flowchart of the enrollment of patients with acute SARS-CoV-2 infection. COVID-19, coronavirus disease 2019; SARS-CoV-2, severe acute respiratory syndrome coronavirus 2; ESRD, end-stage renal disease

During the study period, a total of 592 patients with SARS-CoV-2 infection were included. After excluding 18 patients (one patient with end-stage renal disease and 17 patients whose urine samples were not collected), 574 patients were included in the final analysis. The included patients were then classified into the severe COVID-19 group (all of whom were hospitalized) (*n* = 252; 43.9%), and the non-severe disease group (*n* = 322; 56.1%) (Fig. 1). The overall all-cause mortality rate was 11.8% (68 patients died).

### Comparisons of the severe and non-severe COVID-19 groups

As shown in Table 1, the severe COVID-19 group were older, had a higher percentage of male patients, and had a lower body mass index than the non-severe COVID-19 group (all* p* < 0.05). Overall, 77.5% of the patients did not have a history of smoking, and there was no significant difference in smoking history (current or former) between the two groups. The severe group had significantly higher rates of comorbidities than the non-severe group, except for chronic lung disease.

**Table 1:** Background characteristics and clinical variables of the severe and non-severe COVID-19 patients

Variables	All	Severe	Non-severe	*p* value
	**(*n* = 574)**	**(*n* = 252)**	**(*n* = 322)**	
Age (years)	59.4 ± 18.3	70.9 ± 14.8	50.5 ± 15.6	< 0.001
Male (gender)	306 (53.3%)	167 (66.3%)	139 (43.2%)	< 0.001
Body mass index (kg/m^2^)	24.4 ± 4.9	23.7 ± 4.9	25 ± 4.8	0.002
Smoking history				
Current	52 (9.1%)	27 (10.7%)	25 (7.8%)	0.145
Former	77 (13.4%)	27 (10.7%)	50 (15.5%)	
Never	445 (77.5%)	198 (78.6%)	247 (76.7%)	
Cerebrovascular accident	39 (6.8%)	37 (14.7%)	2 (0.6%)	< 0.001
Hypertension	211 (36.8%)	125 (49.6%)	86 (26.7%)	< 0.001
Diabetes mellitus	147 (25.6%)	105 (41.7%)	42 (13%)	< 0.001
Chronic heart disease	79 (13.8%)	65 (25.8%)	14 (4.3%)	< 0.001
Chronic lung disease	99 (17.2%)	34 (13.5%)	65 (20.2%)	< 0.001
Chronic liver disease	49 (8.5%)	28 (11.1%)	21 (6.5%)	< 0.001
Chronic kidney disease	60 (10.5%)	52 (20.6%)	8 (2.5%)	< 0.001
Immunocompromised status	107 (18.6%)	71 (28.2%)	36 (11.2%)	< 0.001
WBC (10^3^/µL)	9.1 ± 6.7	11.3 ± 9.0	7.3 ± 2.9	< 0.001
Neutrophil (%)	73.2 ± 43.9	83 ± 50.7	65.1 ± 35.4	< 0.001
Lymphocyte (%)	19.8 ± 12.9	10.9 ± 10.4	27.2 ± 9.7	< 0.001
Neutrophil/lymphocyte ratio	3.8 (2–10.3)	10.4 (5.5–19.5)	2.2 (1.7–3.3)	< 0.001
Hemoglobin (g/dL)	12.5 ± 2.4	11.4 ± 2.6	13.3 ± 1.9	< 0.001
Platelets (10^3^/µL)	236.4 ± 97.5	209.4 ± 109.4	258.3 ± 80.6	< 0.001
Serum creatinine (mg/dL)	0.8 (0.7–1.2)	1.1 (0.7–1.8)	0.8 (0.6–0.9)	< 0.001
Ferritin (ng/mL)	378 (150.5–817)	797.5 (439–1239.3)	212 (76.5–381.5)	< 0.001
LDH (U/L)	219.5 (182.8–285)	294 (225.3–382.3)	194 (170.3–225)	< 0.001
CRP (mg/L)	10.9 (1.3–104.5)	106 (35.6–172.5)	1.3 (0.6–3.6)	< 0.001
IL-6 (pg/mL)	6.6 (2.4–40.7)	31.2 (12.1–110)	2.3 (1.6–4.1)	< 0.001
Blood cadmium (μg/L)	0.8 ± 0.5	1.0 ± 0.6	0.7 ± 0.4	< 0.001
Blood nickel (μg/L)	1.5 ± 0.2	1.5 ± 0.3	1.5 ± 0.1	0.320
Urine cadmium/creatinine (μg/g)				
mean ± SD	5.2 ± 19.3	10.5 ± 28.2	0.9 ± 0.8	< 0.001
median (IQR)	1.3 (0.6–3.5)	3.8 (2.2–8.8)	0.7 (0.4–1.1)	< 0.001
Urine nickel (μg/L)				
mean ± SD	2.9 ± 4.3	3.6 ± 6.4	2.4 ± 1.8	0.008
median (IQR)	1.7 (1.4–2.8)	1.8 (1.4–2.9)	1.6 (1.4–2.7)	0.008
Treatment at the time of diagnosis				
Nirmatrelvir and ritonavir (Paxlovid)	55 (9.6%)	21 (8.3%)	34 (10.6%)	< 0.001
Molnupiravir	33 (5.7%)	22 (8.7%)	11 (3.4%)	< 0.001
Remdesivir	193 (33.6%)	193 (76.6%)	0 (0%)	
Dexamethasone	217 (37.8%)	217 (86.1%)	0 (0%)	
Tocilizumab	12 (2.1%)	12 (4.8%)	0 (0%)	
Type of respiratory support				
Room air	322 (56.1%)	0 (0%)	322 (100%)	
Nasal cannula	51 (8.9%)	51 (20.2%)	0 (0%)	
Simple mask	27 (4.7%)	27 (10.7%)	0 (0%)	
High-flow nasal cannula	14 (2.4%)	14 (5.6%)	0 (0%)	
Nonrebreathing mask	11 (1.9%)	11 (4.4%)	0 (0%)	
Invasive mechanical ventilation	149 (26%)	149 (59.1%)	0 (0%)	
Site				
Chang Gung Memorial Hospital Linkou branch	495 (86.2%)	223 (88.5%)	272 (84.5%)	0.165
New Taipei Municipal TuCheng Hospital	79 (13.8%)	29 (11.5%)	50 (15.5%)	0.165
Outpatients	311 (54.2%)	0 (0%)	311 (96.6%)	
Hospitalized	263 (45.8%)	252 (100%)	11 (3.4%)	< 0.001
Hospital mortality	68 (11.8%)	68 (27%)	0 (0%)	

Data are presented as mean ± standard deviation, count (%) or median (interquartile range). *COVID-19* coronavirus disease 2019, *CRP* C-reactive protein, *IL* interleukin, *IQR* interquartile range, *LDH* lactate dehydrogenase, *SD* standard deviation, *WBC* white blood cells

The severe group had significantly higher white blood cell count, neutrophil/lymphocyte ratio, C-reactive protein (CRP) and IL-6 levels than the non-severe group (all* p* < 0.05). Concentrations of cadmium in both blood and urine were significantly higher in the severe group than in the non-severe group (mean value in blood cadmium: 1.0 ± 0.6 μg/L versus 0.7 ± 0.4 μg/L, *p* < 0.001; median value in urine cadmium/creatinine: 3.8 [2.2–8.8] μg/g versus 0.7 [0.4–1.1] μg/g,* p* < 0.001). The concentration of nickel in the blood did not show significant differences between the two groups. The urine nickel concentration was significantly higher in the severe group compared to the non-severe group (median value: 1.8 [1.4–2.9] μg/L versus 1.6 [1.4–2.7] μg/L,* p* = 0.008).

At the time of diagnosis, 193 patients (76.6%) and 217 patients (86.1%) in the severe group received remdesivir and dexamethasone treatment, respectively. More of the patients with severe COVID-19 required oxygen support, 149 patients (59.1%) required invasive mechanical ventilation, and the all-cause hospital mortality rate was 27% (68 patients died).

### Distributions of urine cadmium, severe COVID-19 and hospital mortality

**Fig. 2:**
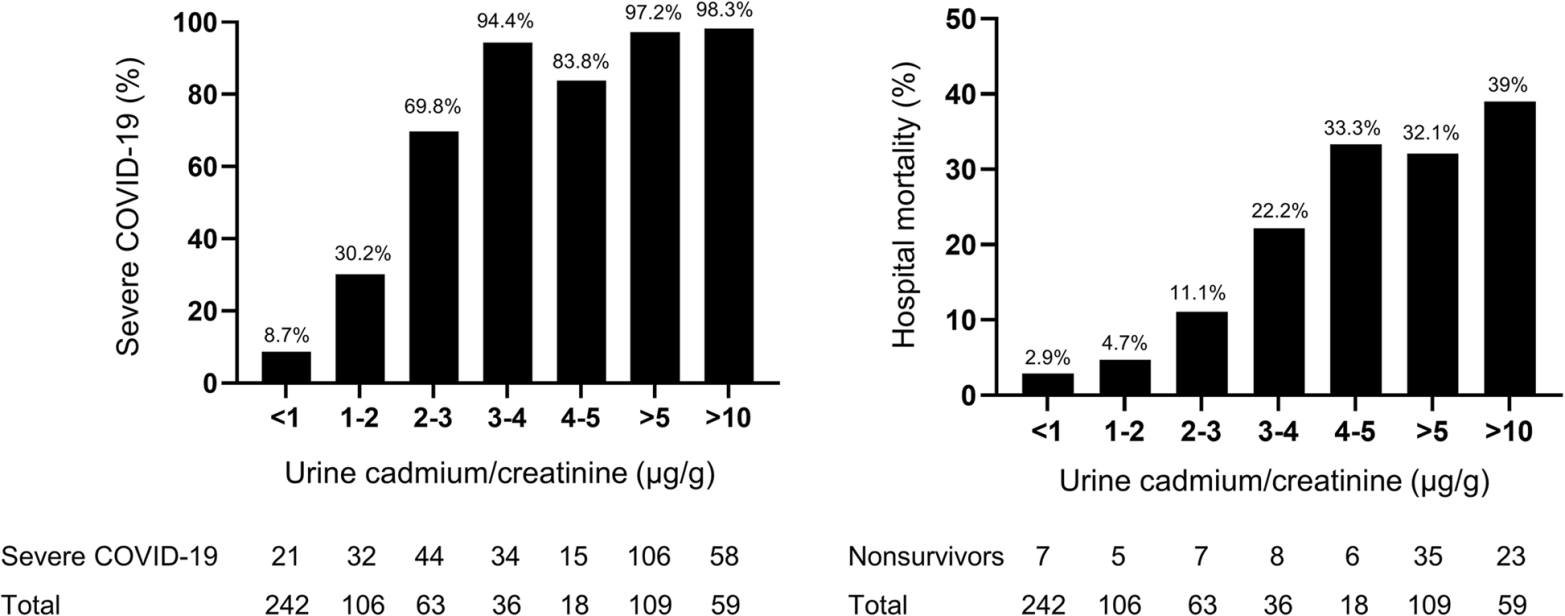
**a** Distribution of urinary cadmium/creatinine values and severe COVID-19. **b** Distribution of urinary cadmium/creatinine values and hospital mortality. COVID-19, coronavirus disease 2019

Overall, 242 patients (42.2%) had a urine cadmium/creatinine value of < 1 μg/g. Of these patients, 21 (8.7%) had severe COVID-19 and 7 (2.9%) died. In addition, 109 patients (19%) had a urine cadmium/creatinine value > 5 μg/g, of whom 106 (97.2%) had severe COVID-19 and 35 (32.1%) died. Moreover, of the 59 patients (10.3%) with a urine cadmium/creatinine value > 10 μg/g, 58 (98.3%) had severe COVID-19 and 23 (39%) died. An increase in urine cadmium concentration was consistently associated with increasing trends in the risk of severe COVID-19 and all-cause hospital mortality except a slight drop in percentage of severe COVID-19 in levels of 4–5 μg/g compared to patients with levels 3–4 μg/g and a slight drop in percentage of death in levels > 5 μg/g compared to patients with levels 4–5 μg/g (Fig. 2).

### Comparisons of the severe COVID-19 patients by quartile of urine cadmium/creatinine

The urine cadmium/creatinine values for each quartile were as follows: quartile 1 (value < 2.2 μg/g), quartile 2 (2.2 ≤ value < 3.8 μg/g), quartile 3 (3.8 ≤ value < 8.8 μg/g), and quartile 4 (value ≥ 8.8 μg/g). As shown in Table 2, patients in the higher urine cadmium/creatinine quartiles were older and had a significantly lower body mass index. No significant differences were observed among the urine cadmium/creatinine quartiles in terms of gender, smoking status, and underlying comorbidities.

**Table 2:** Background characteristics and clinical variables: severe COVID-19 patients as a function of urinary cadmium/creatinine quartiles

Variables	First Quartile	Second Quartile	Third Quartile	Fourth Quartile	*p* value
	**(U-Cd**_**Cr**_** < 2.2 μg/g)**	**(2.2 ≤ U-Cd**_**Cr**_** < 3.8 μg/g)**	**(3.8 ≤ U-Cd**_**Cr**_** < 8.8 μg/g)**	**(U-Cd**_**Cr**_** ≥ 8.8 μg/g)**	
	**(*n* = 63)**	**(*n* = 63)**	**(*n* = 63)**	**(*n* = 63)**	
Age (years)	64.8 ± 16.8	71.8 ± 14.9	73.5 ± 13.4	73.3 ± 12.4	0.002
Male (gender)	46 (73%)	41 (65.1%)	42 (66.7%)	38 (60.3%)	0.425
Body mass index (kg/m^2^)	25.0 ± 5.9	24.9 ± 4.6	22.7 ± 4.5	22.2 ± 4.1	0.001
Smoking history					
Current	9 (14.3%)	7 (11.1%)	7 (11.1%)	4 (6.3%)	0.870
Former	7 (11.1%)	7 (11.1%)	7 (11.1%)	6 (9.5%)	
Never	47 (74.6%)	49 (77.8%)	49 (77.8%)	53 (84.1%)	
Cerebrovascular accident	7 (11.1%)	8 (12.7%)	12 (19%)	10 (15.9%)	0.640
Hypertension	33 (52.4%)	35 (55.6%)	30 (47.6%)	27 (42.9%)	0.465
Diabetes mellitus	27 (42.9%)	27 (42.9%)	25 (39.7%)	26 (41.3%)	0.958
Chronic heart disease	23 (36.5%)	13 (20.6%)	13 (20.6%)	16 (25.4%)	0.110
Chronic lung disease	6 (9.5%)	9 (14.3%)	7 (11.1%)	12 (19%)	0.418
Chronic liver disease	11 (17.5%)	7 (11.1%)	7 (11.1%)	3 (4.8%)	0.149
Chronic kidney disease	14 (22.2%)	13 (20.6%)	14 (22.2%)	11 (17.5%)	0.898
Immunocompromised status	20 (31.7%)	12 (19%)	19 (30.2%)	24 (38.1%)	0.125
APACHE II at diagnosis	14.7 ± 7.8	15 ± 5.6	17.5 ± 5.9	18.9 ± 6.3	0.001
SOFA score at diagnosis	4.8 ± 3.5	5.1 ± 3.3	5.8 ± 3.7	6.4 ± 4.0	0.026
WBC (10^3^/µL)	10 ± 5	11.8 ± 13.1	10.9 ± 6.1	12.3 ± 9.6	0.489
Neutrophil (%)	79.8 ± 13	80.9 ± 13.1	79.2 ± 19.4	81.9 ± 15.4	0.759
Lymphocyte (%)	11 ± 7.5	11.5 ± 12	10.4 ± 9.2	10.8 ± 12.4	0.943
Neutrophil/lymphocyte ratio	9.5 (5–16)	9.8 (5.8–15.8)	10.3 (4.7–19.5)	12.8 (6.8–26.7)	0.02
Hemoglobin (g/dL)	11.6 ± 2.7	12 ± 2.4	10.9 ± 2.5	11.3 ± 2.6	0.098
Platelets (10^3^/µL)	203.6 ± 92.4	215.8 ± 105.5	187.9 ± 105.8	230.7 ± 128	0.155
Serum creatinine (mg/dL)	1.3 (0.8–1.9)	1.1 (0.7–1.5)	1.1 (0.7–1.9)	1 (0.6–1.6)	0.252
Ferritin (ng/mL)	707 (352.5–1026)	680 (403–976.5)	985 (650.8–1221.3)	1119 (526–1881)	0.007
LDH (U/L)	262 (203.5–334)	271 (215–354)	320 (248.3–392.5)	343 (263.8–431.3)	0.548
CRP (mg/L)	54 (13.3–169.5)	108.5 (38.9–155.3)	123.2 (52.6–157.9)	126.2 (60.9–175.6)	0.139
IL-6 (pg/mL)	6 (18.3–56.7)	15.1 (6.5–42.1)	48 (19–97.7)	59.8 (23.2–196.5)	0.324
Blood cadmium (μg/L)	0.7 ± 0.3	0.9 ± 0.4	1.0 ± 0.6	1.3 ± 0.8	< 0.001
Blood nickel (μg/L)	1.5 ± 0.1	1.6 ± 0.6	1.5 ± 0.1	1.5 ± 0.1	0.446
Urine cadmium/creatinine (μg/g)					
mean ± SD	1.2 ± 0.6	2.9 ± 0.4	6 ± 1.5	31.9 ± 50.9	< 0.001
median (IQR)	1.3 (0.7–1.7)	2.9 (2.6–3.3)	6 (4.8–6.8)	16.5 (11.7–27.4)	< 0.001
Urine nickel (μg/L)					
mean ± SD	2.7 ± 2.5	3.9 ± 8.4	4.2 ± 7.6	3.6 ± 5.0	0.661
median (IQR)	1.5 (1.4–2.7)	1.7 (1.4–3.0)	2.1 (1.4–3.1)	1.9 (1.4–2.9)	0.661
PaO_2_/FiO_2_ (mm Hg)	220.8 (136–287.7)	149 (103.8–240.2)	147.2 (97.8–203.7)	134 (103.8–204.3)	0.032
Type of respiratory support					
Nasal cannula	23 (36.5%)	18 (28.6%)	5 (7.9%)	5 (7.9%)	< 0.001
Simple mask	5 (7.9%)	8 (12.7%)	10 (15.9%)	4 (6.3%)	0.310
High-flow nasal cannula	4 (6.3%)	4 (6.3%)	5 (7.9%)	1 (1.6%)	0.443
Nonrebreathing mask	2 (3.2%)	2 (3.2%)	5 (7.9%)	2 (3.2%)	0.486
Invasive mechanical ventilation	29 (46%)	31 (49.2%)	38 (60.3%)	51 (81%)	< 0.001

Data are presented as mean ± standard deviation, count (%) or median (interquartile range)

*APACHE* Acute Physiology and Chronic Health Evaluation, *COVID-19* coronavirus disease 2019, *CRP* C-reactive protein, *FiO*_*2*_ fraction of inspired oxygen, *IL* interleukin, *IQR* interquartile range, *LDH* lactate dehydrogenase, *PaO*_*2*_ partial pressure of oxygen in arterial blood, *SD* standard deviation, *SOFA* Sequential Organ Failure Assessment, *U-Cd*_*Cr*_ urinary cadmium/creatinine

APACHE II and SOFA scores at the diagnosis of acute SARS-CoV-2 infection were significantly different among quartiles, and both showed a stepwise increasing trend with an increase in urine cadmium/creatinine quartile (*p* = 0.001 and 0.026, respectively). Neutrophil/lymphocyte ratio and ferritin were significantly higher in the higher urine cadmium/creatinine quartiles.

The values of CRP and IL-6 also revealed a stepwise increasing trend with an increase in urine cadmium/creatinine quartile, although the differences among the quartiles did not reach significance. The patients in the higher urine cadmium/creatinine quartiles tended to have higher risks of hypoxemia (i.e. lower PaO_2_/FiO_2_,* p* = 0.032) and respiratory failure requiring invasive mechanical ventilation (*p* < 0.001).

### Clinical outcomes of the severe COVID-19 patients by quartile of urine cadmium/creatinine

As shown in Table 3, there were significantly higher 60-, 90-day, and all-cause hospital mortality rates in the higher urine cadmium/creatinine quartiles (*p* = 0.034, 0.023 and 0.021, respectively). Both ICU admission rate and length of ICU stay were significantly higher in the higher urine cadmium/creatinine quartiles, whereas there were no significant differences in the duration of mechanical ventilation and length of hospital stay among the quartiles.

**Table 3:** Clinical outcomes as a function of urinary cadmium/creatinine quartiles in the severe COVID-19 patients

Variables	First Quartile	Second Quartile	Third Quartile	Fourth Quartile	*p* value
	**(U-Cd**_**Cr**_** < 2.2 μg/g)**	**(2.2 ≤ U-Cd**_**Cr**_** < 3.8 μg/g)**	**(3.8 ≤ U-Cd**_**Cr**_** < 8.8 μg/g)**	**(U-Cd**_**Cr**_** ≥ 8.8 μg/g)**	
	**(*n* = 63)**	**(*n* = 63)**	**(*n* = 63)**	**(*n* = 63)**	
Mortality					
28-day hospital mortality	6 (9.5%)	5 (7.9%)	8 (12.7%)	12 (19%)	0.244
60-day hospital mortality	11 (17.5%)	8 (12.7%)	14 (22.2%)	21 (33.3%)	0.034
90-day hospital mortality	12 (19%)	9 (14.3%)	17 (27%)	23 (36.5%)	0.023
All cause hospital mortality	13 (20.6%)	12 (19%)	17 (27%)	26 (41.3%)	0.021
Shock status	24 (38.1%)	21 (33.3%)	24 (38.1%)	37 (58.7%)	0.019
Inotropic agents use	19 (30.2%)	18 (28.6%)	24 (38.1%)	37 (58.7%)	0.002
Acute kidney injury	32 (50.8%)	24 (38.1%)	27 (42.9%)	24 (38.1%)	0.375
Renal replacement therapy	12 (19%)	10 (15.9%)	5 (7.9%)	9 (14.3%)	0.305
ARDS	23 (36.5%)	28 (44.4%)	31 (49.2%)	42 (66.7%)	0.008
ICU admission	37 (58.7%)	36 (57.1%)	45 (71.4%)	54 (85.7%)	0.002
Duration of mechanical ventilator (days)	0 (0–11.8)	4 (0–14)	6 (0–20)	11 (5–26)	0.184
Length of ICU stay (days)	7.5 (0–17.5)	7 (0–16.5)	11.5 (0–22)	16 (8.5–34)	0.025
Length of hospital stay (days)	19.5 (11.3–37.3)	21 (12–35)	25 (16–39.3)	37 (20–65.5)	0.107

Data are presented as mean ± standard deviation, count (%) or median (interquartile range). *ARDS* acute respiratory distress syndrome, *COVID-19* coronavirus disease 2019, *ICU* intensive care unit, *U-Cd*_*Cr*_ urinary cadmium/creatinine

There were also no significant differences among the quartiles regarding new-onset acute kidney injury and renal replacement therapy. Patients in the higher urine cadmium/creatinine quartiles were significantly associated with shock status, use of inotropic agents, and the occurrence of ARDS (all *p* < 0.05).

### Factors associated with severe COVID-19

After adjusting for significant confounding variables, multivariable logistic regression models revealed that the following factors were independently positively associated with severe COVID-19: lower lymphocyte count, lower platelet count, higher values of lactate dehydrogenase (LDH), CRP, and urine cadmium/creatinine. The patients who had higher urine cadmium/creatinine values had a significantly higher odds of severe COVID-19 (adjusted OR 1.643, [95% CI 1.060–2.547], *p* = 0.026). The maximum Youden index value was used to categorize all of the enrolled patients into those with high or low urine cadmium/creatinine levels at the diagnosis of acute SARS-CoV-2 infection, with a cutoff value of 2.05 μg/g (area under the ROC curve 0.911, [95% CI 0.885–0.937], *p* < 0.001). A urine cadmium/creatinine value > 2.05 μg/g had the highest predictive value among all studied variables and was independently associated with severe COVID-19 (adjusted OR 5.349, [95% CI 1.118–25.580], *p* = 0.036) (Table 4).

**Table 4:** Multivariable logistic regression analysis of factors associated with severe COVID-19

Variables	Univariable analysis		Multivariable analysis model 1		Multivariable analysis model 2	
	**OR (95% CI)**	***p* value**	**Adjusted OR (95% CI)**	***p* value**	**Adjusted OR (95% CI)**	***p* value**
CGMH linkou branch	1.414 (0.865–2.309)	0.167				
Age (with each year increase)	1.088 (1.072–1.103)	< 0.001				
Male	2.587 (1.838–3.641)	< 0.001				
Body mass index	0.947 (0.914–0.980)	0.002				
Smoking (current)	1.426 (0.805–2.523)	0.223				
Smoking (former)	0.653 (0.396–1.077)	0.095				
≥ One comorbidity	7.359 (4.668–11.601)	< 0.001				
WBC	1.219 (1.158–1.283)	< 0.001				
Neutrophil	1.064 (1.049–1.078)	< 0.001				
Lymphocyte	0.857 (0.836–0.878)	< 0.001	0.860 (0.787–0.940)	0.001	0.888 (0.838–0.942)	< 0.001
Neutrophil/lymphocyte ratio	1.272 (1.208–1.338)	< 0.001				
Hemoglobin	0.695 (0.639–0.757)	< 0.001				
Platelets	0.994 (0.992–0.996)	< 0.001	0.984 (0.973–0.995)	0.005	0.986 (0.977–0.995)	0.004
Serum creatinine	2.835 (2.033–3.955)	< 0.001				
Ferritin	1.003 (1.002–1.004)	1.003				
LDH	1.017 (1.013–1.020)	< 0.001	1.010(1.001–1.020)	0.035	1.014 (1.004–1.024)	0.005
CRP	1.107 (1.080–1.134	< 0.001	1.045 (1.013–1.077)	0.005	1.060 (1.031–1.090)	< 0.001
IL-6	1.184 (1.126–1.246)	< 0.001				
Blood cadmium	3.220 (2.075–4.996)	< 0.001				
Blood nickel	1.575 (0.588–4.222)	0.367				
Urine cadmium/creatinine	3.780 (2.950–4.844)	< 0.001	1.643 (1.060–2.547)	0.026		
Urine nickel	1.091 (1.023–1.164)	0.008				
Urine cadmium/creatinine > 2.05 μg/g					5.349 (1.118–25.580)	0.036

*CGMH* Chang Gung Memorial Hospital, *CI* Confidence interval, *COVID-19* Coronavirus disease 2019, *CRP* C-reactive protein, *IL* Interleukin, *LDH* Lactate dehydrogenase, *OR* Odds ratio, *WBC* White blood cells

For the continuous variables, the odds ratio means that the odds of severe COVID-19 increases or decreases per unit increase of these variables

Model 1: add urine cadmium/creatinine and urine nickel as continuous variables

Model 2: add urine cadmium/creatinine > 2.05 μg/g as a categorical variable

## Discussion

The key finding of this study is that urine cadmium had the greatest predictive value for disease severity among all studied clinical variables in the patients with COVID-19 who had no recent or chronic history of exposure to cadmium.

Oxidative stress plays a crucial role in the pathophysiology of SARS-CoV-2 infection. Endothelial oxidative stress causes a reduction in vascular nitric oxide bioavailability, and this effect is proportional to the severity of COVID-19 and may participate in the pathogenesis of microvascular endothelial damage, cytokine storm, immunothrombosis, microangiopathy, and multi-organ injury during SARS-CoV-2 infection [26–28].

A previous review article demonstrated that toxic metal exposure including arsenic, cadmium, mercury, and lead is associated with reduced lung function and respiratory diseases (chronic obstructive pulmonary disease and bronchitis). These associations may be related to airway inflammation, increased ROS generation, oxidative stress, and apoptosis induced by heavy metal exposure [7]. A retrospective study showed that urinary concentrations of chromium, manganese, copper, selenium, cadmium, mercury and lead adjusted by urinary creatinine were higher in severe COVID-19 patients than the non-severe COVID-19 patients. Among the severe COVID-19 patients, these urinary trace elements were also higher in the deceased group compared to the recovered group [32]. Another study found that blood cadmium level was higher in the deceased group than the recovered group among severe COVID-19 patients [33].

Cadmium exposure has been reported to alter reduction–oxidation (i.e., redox) balance and subsequently induce ROS overproduction, trigger oxidative stress and mitochondrial electron transport chain dysfunction, stimulate endoplasmic reticulum stress signaling pathways with ensuing apoptotic cell death, and thereby alter inflammatory responses and inhibit immune function [6, 16, 18–20, 34]. Nickel can impair immune system function by causing oxidative stress, mitochondrial damage, and apoptosis. Furthermore, the accumulation of nickel can activate inflammation, increase cytokines secretion, and induce immunotoxicity [23–25]. In addition to cadmium and nickel exposure, air pollutants such as particulate matter smaller than 2.5 μm in diameter (PM 2.5) and ozone, along with environmental chemicals like perfluorinated alkylates, can induce oxidative stress, systemic inflammation and exert an immunosuppressive effect that could deteriorate the course of infectious diseases [35–37].

The potential impact and correlation of cadmium and nickel with disease severity and clinical outcomes of COVID-19 have not been clearly elucidated. In the current study, we found that the severe COVID-19 group had significantly higher values of blood and urine cadmium, significantly higher values of urine nickel, significantly higher neutrophil/lymphocyte ratio, significantly higher values of inflammatory markers including ferritin, LDH, CRP and IL-6, and higher rate of oxygen support than the non-severe COVID-19 group. These findings suggest that cadmium or nickel exposure may worsen or aggravate the harmful effects of oxidative stress and dysregulated inflammatory or immune responses induced by SARS-CoV-2 infection, thereby further contributing to increased inflammation, impaired gas exchange, defective cell and tissue damage, and pulmonary and systemic organ injury, and consequently the severity of COVID-19. Cadmium is a cumulative toxin, with its concentration in the body increasing over time due to slow elimination. In contrast, nickel is not a cumulative toxin. Therefore, it is reasonable to assume that cadmium, accumulating in the body over a lifetime, may predispose COVID-19 patients to more serious illness compared to nickel. In our multivariable regression model, urine cadmium/creatinine had a higher predictive value than urine nickel and remained independently associated with severe COVID-19 (adjusted OR 1.643, *p* = 0.026).

Among the severe COVID-19 patients, those in the higher urinary cadmium quartiles had a significantly higher neutrophil/lymphocyte ratio, higher levels of inflammatory markers including ferritin, LDH, IL-6 and CRP, significantly higher risk of organ failure (i.e., higher APACHE II and SOFA scores), and significantly higher risk of hypoxemia (i.e., lower PaO_2_/FiO_2_) requiring a significantly higher rate of invasive mechanical ventilation. In addition, the severe COVID-19 patients in the higher urine cadmium quartiles had worse clinical outcomes, including a significantly higher risk of shock status requiring the use of inotropic agents, significantly higher risk of ARDS, and significantly higher 60-day, 90-day, and all-cause hospital mortality rates (all *p* < 0.05). These findings suggest that a higher urine cadmium concentration, representing a higher total body burden of cadmium, may increase oxidative stress, promote excessive inflammation and immune responses, augment cytokine production, impair oxygen exchange, and cause distant organ damage. This may then predispose COVID-19 patients to serious complications, worse clinical outcomes and even death.

Long-term or chronic exposure to cadmium, even at lower levels, is carcinogenic to humans and can damage multiple systems, especially the kidneys, bones, and lungs. Long-term cadmium accumulation primarily occurs in the kidneys [13]. The half-life of blood cadmium is between 75 and 128 days, while the average half-life of cadmium in the kidneys ranges from 6 to 38 years [13, 14, 17]. Therefore, urinary cadmium value is a better surrogate of lifetime accumulation or long periods of exposure, total body burden and renal accumulation of cadmium, whereas the blood concentration of cadmium reflects acute or recent exposure [13, 14, 38].

The upper limit threshold for the value of urinary cadmium is lacking for many clinical diseases, including COVID-19 [13, 39]. The Food and Agriculture Organization and World Health Organization established a urinary cadmium threshold of 5.24 μg/g creatinine. However, some cohort studies have reported that a urinary cadmium threshold value below 5.24 μg/g creatinine was associated with adverse health effects such as an increased risk of type 2 diabetes, cardiovascular disease, chronic kidney disease and cancer, and that this threshold limit should be reassessed [12, 13, 40]. In the current study, the risks of severe COVID-19 and all-cause hospital mortality demonstrated stepwise increasing trends with an increase in urine cadmium/creatinine value. In multivariable regression models, urine cadmium/creatinine remained independently associated with severe COVID-19 (adjusted OR 1.643, *p* = 0.026), and a value > 2.05 μg/g had the highest predictive value among all studied clinical variables (adjusted OR 5.349, *p* = 0.036), indicating that individuals with a higher urine cadmium concentration, reflecting higher total body burden or long-term exposure to cadmium, may be more vulnerable to severe COVID-19 and subsequently poor clinical outcomes and higher in-hospital mortality.

Our findings indicate that specific attention should be paid to individuals with risk factors to prevent serious complications of COVID-19. Previous studies have reported that lymphopenia, thrombocytopenia, and elevated values of LDH and CRP were all associated with increased severity and mortality in COVID-19 patients [3, 4, 41–43]. Hematological changes such as lymphopenia and thrombocytopenia are not rare and have been reported in up to 80% and 40% of patients with COVID-19, respectively. SARS-CoV-2 may directly infect lymphocytes, destroy lymphatic organs, and cause T cell exhaustion. Inflammatory cytokine-induced lymphocyte apoptosis and suppression of lymphocyte proliferation due to coexisting metabolic disorders (i.e., lactic acidosis) have also been associated with lymphopenia in patients with COVID-19 [41, 44]. The possible mechanisms of SARS-CoV-2 infection-induced thrombocytopenia include direct hematopoietic stem or progenitor cell invasion and lung injury mediated by autoantibodies and immune complexes, defective bone marrow microenvironment, decreased thrombopoietin production, and inhibition of megakaryocytopoiesis by cellular immunity and cytokine storm [45, 46]. Markedly higher levels of inflammatory indicators such as LDH and CRP have been reported in patients with severe COVID-19 compared to those with non-severe COVID-19, and this has been associated with higher risks of ARDS, ICU admission, and death [3, 41, 43, 47], similar to the results of our study. In the current study, besides urine cadmium, we found that lymphopenia, thrombocytopenia, elevated LDH, and elevated CRP were all independently associated with severe COVID-19 in the multivariable regression models (all *p* < 0.05). This finding suggests that excessive inflammation and immune suppression upon sepsis caused by SARS-CoV-2 infection may influence the severity and clinical outcomes of COVID-19.

A previous study reported that advanced age and comorbidities were associated with increased risk of death in COVID-19 [48]. Cadmium accumulates with age, and elderly patients could have higher total body burden of cadmium which may deteriorate the disturbed oxidative stress during SARS-CoV-2 infection and succumb to serious illness of COVID-19. Our study observed that age, underlying comorbidities, and blood and urine cadmium levels of severe COVID-19 patients were all significantly higher than those with non-severe COVID-19. In our multivariable models, urine cadmium/creatinine remained independently associated with severe COVID-19.

There are several limitations to this study. First, although this cohort study was conducted at two medical centers in Taiwan, it may not be generalizable to other institutions. Second, the vaccination status was not completely recorded and SARS-CoV-2 variants were not checked in all individuals, although the omicron variant was predominant during the study period. Third, although cadmium concentration in a single spot urine specimen has been reported to be an indicator of long-term exposure to cadmium [10], we only collected blood and urine samples once at the diagnosis of acute SARS-CoV-2 infection, and so serial changes or dynamic monitoring of cadmium values was not possible. To adjust for variations in urine dilution or hydration, we use urinary creatinine-corrected levels of urinary cadmium, but the urinary nickel was not routinely corrected by urinary creatinine in these two medical centers. Finally, our objective in this observational study was to identify associations between blood or urine cadmium and nickel and clinical outcomes of COVID-19 patients without considering issues pertaining to causality. We did not examine the biological effect of cadmium and nickel on the pathophysiology of SARS-CoV-2 infection, and the exact cellular mechanism is still unclear.

## Conclusions

Our findings revealed that urinary cadmium concentration at the diagnosis of acute SARS-CoV-2 infection was significantly associated with the severity and clinical outcomes of COVID-19 patients, and that it may predispose COVID-19 patients to develop severe complications and even death. Further investigations are warranted to verify the causal relationship and whether urinary cadmium level in the early course of COVID-19 could be regarded as a valuable marker to predict the severity and outcomes of patients with COVID-19.

### Supplementary Information

**Supplementary Material 1.**

## Data Availability

The datasets used or analyzed in the study are available from the corresponding author on reasonable request.

## Abbreviations

APACHE: Acute physiology and chronic health evaluation
ARDS: Acute respiratory distress syndrome
CI: Confidence interval
COVID-19: Coronavirus disease 2019
CRP: C-reactive protein
ICU: Intensive care unit
IL: Interleukin
LDH: Lactate dehydrogenase
OR: Odds ratio
ROC: Receiver operating characteristic
ROS: Reactive oxygen species
SARS-CoV-2: Severe acute respiratory syndrome coronavirus 2
SOFA: Sequential organ failure assessment
