# Supplementary material for: Urinary cadmium concentration is associated with the severity and clinical outcomes of COVID-19: a bicenter observational cohort study
Source: Environ Health. 2024 Mar 19;23:29. doi: 10.1186/s12940-024-01070-6 (PMC10949676; doi:10.1186/s12940-024-01070-6)
Supplement: Supplementary file 1 — Supplementary Material 1. [file 12940_2024_1070_MOESM1_ESM.docx]

**Supplementary Information**

**Urinary cadmium concentration is associated with the severity and clinical outcomes of COVID-19: a bicenter observational cohort study**

**Authors:** Li-Chung Chiu, Chung-Shu Lee, Ping-Chih Hsu, et al.

**Measurements of blood cadmium concentration**

Blood cadmium measurements were conducted using inductively coupled plasma mass spectrometry (ICP-MS). Blood specimens were collected in 6 mL plastic blood collection tubes containing K2EDTA as an anticoagulant (BD, Franklin Lakes, NJ, USA), and stored at 4°C. Cadmium concentration was quantified using ICP-MS on a PerkinElmer NexION 350X instrument (Waltham, MA, USA), and analyzed using no-gas mode. Blood specimens (500 μL) were diluted 10 times with 1.5% (*w/v*) nitric acid (JT Baker, Phillipsburg, NJ, USA) solution containing yttrium as an internal standard. The cadmium and yttrium standards were purchased from AccuStandard (New Haven, CT, USA). A standard calibration curve with a range of 0 to 40 μg/L was created. The calibration curve had a correlation coefficient of more than 0.995. Level 1 control in-house prepared control and level 2 control Seronorm trace elements whole blood control (Sero, Billingstad, Norway) were used and analyzed at the start and end of each analytical run and after every 10 samples. The lower limit of quantitation (LOQ) for blood cadmium with ICP-MS was 0.5 μg/L. Therefore, values below 0.5 μg/L were assigned to this LOQ (i.e., 0.5 μg/L) for analysis.

**Measurements of urine cadmium concentration**

Urinary cadmium measurements were also conducted using ICP-MS. Urine specimens were collected in 10 mL metal-free plastic collection tubes and stored at 4°C. Urinary cadmium concentration was quantified using ICP-MS on an Agilent 7800 ICP-MS instrument (Santa Clara, CA, USA) and analyzed using no-gas mode. Urine specimens (500 μL) were diluted 10 times with 1.5% (*w/v*) nitric acid (JT Baker, Phillipsburg, NJ, USA) solution containing yttrium as an internal standard. The standard range was 2.67 to 355.84 nmol/L. The calibration curve had a correlation coefficient of more than 0.995. BIO-RAD Lyphochek® Urine Metal Control Levels 1, 2, and 3 (Hercules, CA, USA) were used and analyzed at the beginning and end of each analytical run; they were then analyzed again after every 10 samples. The lower LOQ for urinary cadmium was 2.67 nmol/L, and values below the LOQ were assigned to this LOQ for analysis. Each urinary cadmium result was corrected using the urinary creatinine level of the same urine sample.

**Measurements of blood nickel concentration**

Blood nickel measurements were conducted using ICP-MS. Blood specimens were collected in 6 mL plastic blood collection tubes containing K2EDTA as an anticoagulant (BD, Franklin Lakes, NJ, USA), and stored at 4°C. The nickel ions were quantified by a PerkinElmer NexION 350X instrument (Massachusetts, USA). The analysis process was performed using a dynamic reaction cell with methane to eliminate polyatomic inter­ferences. Blood specimens (500 μL) were diluted 10 times with 1.5% (*w/v*) nitric acid (JT Baker, Phillipsburg, NJ, USA) solution containing yttrium as an internal standard. The standards were obtained from AccuStandard (Connecticut, USA). Six calibration points were made by spiking the standard into blank urine to give nickel concentrations of 1.25, 2.5, 5, 10, 20, and 40. Calibration was performed after reagent blank, blank urine and the six calibration standards in the internal standard diluent solution. The calibration curve had an R ≥ 0.995. Lypocheck urine metals control level 1 and level 2 (Bio–Rad Laboratories, Hemel Hempstead, UK) as internal quality controls were examined at the start and end of each analytical run and again after every ten samples. The lower limit of quantitation for blood nickel was 1.4 μg/L, and values below the limit of quantitation were allocated to the limit of quantitation for analysis.

**Measurements of urine nickel concentration**

Urinary nickel measurements were also conducted using ICP-MS. The urine samples were collected in 10 mL metal-free plastic collection tubes. The nickel ions were quantified by a PerkinElmer NexION 350X instrument (Massachusetts, USA). The analysis process was performed using a dynamic reaction cell with methane to eliminate polyatomic inter­ferences. Urine specimens (500 μL) were diluted (1 + 9) with a 1.5% nitric acid (JT Baker, New Jersey, USA) solution containing yttrium as an internal standard. The standards were obtained from AccuStandard (Connecticut, USA). Six calibration points were made by spiking the standard into blank urine to give nickel concentrations of 1.25, 2.5, 5, 10, 20, and 40. Calibration was performed after reagent blank, blank urine and the six calibration standards in the internal standard diluent solution. The calibration curve had an R ≥ 0.995. Lypocheck urine metals control level 1 and level 2 (Bio–Rad Laboratories, Hemel Hempstead, UK) as internal quality controls were examined at the start and end of each analytical run and again after every ten samples. The lower limit of quantitation for urine nickel was 1.4 μg/L, and values below the limit of quantitation were allocated to the limit of quantitation for analysis.
